# Pupil size variation as a response to stress in European catfish and its application for social stress detection in albino conspecifics

**DOI:** 10.1371/journal.pone.0244017

**Published:** 2020-12-31

**Authors:** Ondřej Slavík, Pavel Horký, Josef Velíšek, Tereza Valchářová

**Affiliations:** 1 Department of Zoology and Fisheries, Faculty of Agrobiology, Food and Natural Resources, Czech University of Life Sciences, Prague, Czech Republic; 2 South Bohemian Research Centre of Aquaculture and Biodiversity of Hydrocenoses, Faculty of Fisheries and Protection of Waters, University of South Bohemia in České Budějovice, Vodňany, Czech Republic; Xiamen University, CHINA

## Abstract

Hormonal changes such as increased cortisol level in blood plasma in response to stress and social environmental stimuli are common among vertebrates including humans and typically accompanied by other physiological processes, such as changes in body pigmentation and/or pupil dilatation. The role of pupil size variation (PSV) as a response to stress have yet to be investigated in fish. We exposed albino and pigmented European catfish to short-term stress and measured changes in pupil size and cortisol level. Albinos showed lower pupil dilatation and higher cortisol levels than did pigmented conspecifics. A clear positive relationship between pupil dilatation and cortisol concentrations was observed for both pigmented and albino specimens, suggesting that PSV can be used as a stress indicator in fish, irrespective of albino’s inability to express social communication by coloring. During the follow-up, we investigated whether a penultimate contest between albino individuals would impact contestants’ social stress during subsequent contact. We observed PSV during the contact of unfamiliar albino catfish with different penultimate experiences (winner (W) and/or loser (L)). Then, the following treatment combinations were tested: WW, WL and LL. Twenty-four-hour contact of two unfamiliar catfish resulted in higher pupil dilatation among individuals with previous winner experience. Among treatment combinations, a WL contest displayed the highest pupil dilatation for winners. PSV reflected socially induced stress in individuals that was accompanied by the “winner” experience and dominancy in albinos. To conclude, the present study validates pupil dilatation as a non-invasive method to evaluate stress level in pigmented as well as albino fish in various contexts.

## Introduction

In fish, physiological responses to changes in the social environment involve changes in hormonal activity, including cortisol level, that are accompanied by other changes, such as changes in body pigmentation and eye darkening. Cortisol production in fish is reportedly associated with negative stimuli and stress [1–3]. Cortisol level reflects the status of an individual in the social hierarchy [4, 5] and tends to be higher in subordinate individuals and individuals under chronic stress [5, 6]. More aggressive individuals display lower responsiveness to stress and produce less cortisol than do less aggressive individuals [7]. Cortisol concentrations in blood plasma are associated with levels of body pigmentation. In salmonids, more pigmented dominant individuals exhibit more intense and frequent spots than do subordinate individuals and they also display lower responsiveness to stress and lower cortisol levels [8, 9]. However, in the Arctic charr *Salvelinus alpinus*, more stressed individuals have more carotenoid spots, whilst cortisol levels in blood plasma are lower in dominant charr being positively correlated with frequency of aggressive attacks by dominant individuals [10].

However, albinos cannot express social communication by variability in coloring. Albinism is generally the result of combinations of homozygous recessive mutations from pigmented parents, and in particular, albinos are often unable to synthesize tyrosine and melatonin hormones. This disability is associated not only with red irises and light skin coloring (oculocutaneous albinism, OCA; [11]) but also with physiological, behavioral and social alterations. For example, compared to pigmented conspecifics, albinos are less active and aggressive [12–14] and albinos are socially ostracized [15], which can explain why they are presented as losers in competition for resources [16]. Hence, for detecting the stress-induced status of an albino individual accompanying its social interactions, it is not possible to use the methods based on variability in pigmentation and/or eye darkening reported for salmons [17], tropical cichlids and cyprinids [18–21]. Presumably, stress in albinos can be alternatively detected by pupil size variation (PSV). While pupil modulation occurs in response to illumination, a process that is well understood [22], it can also occur in association with various cognitive processes [23]. The diameter of the pupil is primarily controlled by the iris sphincter muscle, which constricts the pupil, and by the dilatory pupillary muscle, which promotes pupil dilatation [24]. Although both muscles are under the control of the parasympathetic and sympathetic nervous systems, sympathetic pathways primarily impend dilatation in response to arousal and not luminescence [25]. Pupil dilatation is associated with danger perception, stress [26, 27] and discomfort, such as pain [28, 29], sleep deprivation [30] and negative visual stimuli [31, 32], and has widely been used as an indicator of emotional arousal in humans [33, 34]. Furthermore, several recent animal studies have revealed that non-luminance-induced changes in pupil size can be used to track the fluctuation of cortical arousal and cognitive factors (for a review, see [35]), suggesting that the correlation between pupil size and behavioral state is a universal phenomenon across mammalian species [36]. Fluctuating arousal state has been shown to modulate pupil changes in association with perceptual performance and reaction time in rats [36], and pupil-size changes in monkeys have been shown to be evoked by both the social environment and arousal [37].

The cortisol concentration in blood plasma is often used as a physiological stress indicator [38], and an increased level is related to stress-induced pupil dilatation [39]. As reported for humans and some other mammal species, pupil size can be associated with socially induced stress [37] and cortisol levels in blood plasma [39]. These relationships have yet to be investigated in fish. The aims of our study were I) to test if there is a relationship between PSV and cortisol levels in pigmented as well as albino fish under a standardized acute stress exposure [40, 41] in a methodological experiment and II) to test the application of the PSV methodology under more complex socially induced stress [42–44] in albinos that have several physiological, behavioral and social alterations when compared to pigmented conspecifics [11–16]. In experiment II, we used the winner-loser test in which the previous winning experience of an individual strengthens its will to contest and increases the probability of its subsequent victory [45–47]. We observed a difference between socially induced stress of a winner and/or loser of albino fish based exclusively on noninvasive PSV that is more appropriate with regard to animal welfare then blood sampling.

We used albino and pigmented European catfish *Silurus glanis* (Linnaeus, 1758), a freshwater predator achieving a size of 2.7 m and a weight up to 130 kg with high invasion potential in areas of nonnative occurrence [48], as experimental animals. Catfish are commonly hatchery-reared species [49], and juveniles are often used in laboratory experiments focusing on social and diurnal behavior, food intake, and/or energy consumption [50, 51]. Juveniles compete for food and space, and when shelters are offered, individuals fight for them [52]. Albino individuals occur in both wild and aquaculture environments [15, 53–55].

## Materials and methods

### Ethics statement

All of the laboratory experimental procedures complied with valid legislative regulations (Law no. 246/1992, § 19, art. 1, letter c), which derived from the Directive 2010/63/EU; the permit was subjected to O. Slavík, qualified according to according to Law no. 246/1992, § 17, art. 1; permit no. CZ00167. All laboratory sampling including PIT implantation was carried out with the relevant permission from the Departmental Expert Committee for authorization experimental project of the Ministry of Education, Youth and Sports of the Czech Republic (permit no. MSMT– 1972/2016-5). The maintenance staff has been trained by law in animal care to maintain the high quality of the experiment. The number of experimental animals and all methods used comply with the reduction, replacement, and refinement of animal experimentation. The study did not involve endangered species.

### Experimental animals

Two groups of juvenile European catfish from the Znojmo hatchery, Czech Republic, were transported into the laboratory one month prior to each experiment. Both groups, the group of pigmented individuals (200 individuals) and the group of albinos (200 individuals), were from different pigmented parents. In the laboratory, experimental animals were further divided into eight groups (4 albino and 4 pigmented groups), containing fifty individuals each, that were kept in separate tanks (each tank had a volume of 380 l) for 1 month. All fish were PIT tagged. To facilitate visual orientation among storage tanks, we also marked individuals using visible implant elastomer (VIE) tags (Northwest Marine Technology, USA). Fish were maintained under standard conditions with the aim to maximize their welfare [56]. Water was refined using biological filters with an integrated UV sterilizer (Pressure-Flo 5000, Rolf C. Hagen Inc., www.lagunaponds.com). The water temperature was controlled automatically using external air conditioning and held at an average of 20°C. Light was controlled under a 12-h day/12-h night regime. The fish were fed *ad libitum* with Biomar pellets (Czech Republic) once a day. Shelters (one shelter per fish; plastic tubes; diameter 5 cm; length adjusted to the size of the fish) were installed in each tank [52]. All experimental fish survived. After the experiment, the fish were released under the control of the Fish Management Authorities into fishponds with extensive production management.

### Experimental design

**Fig 1:**
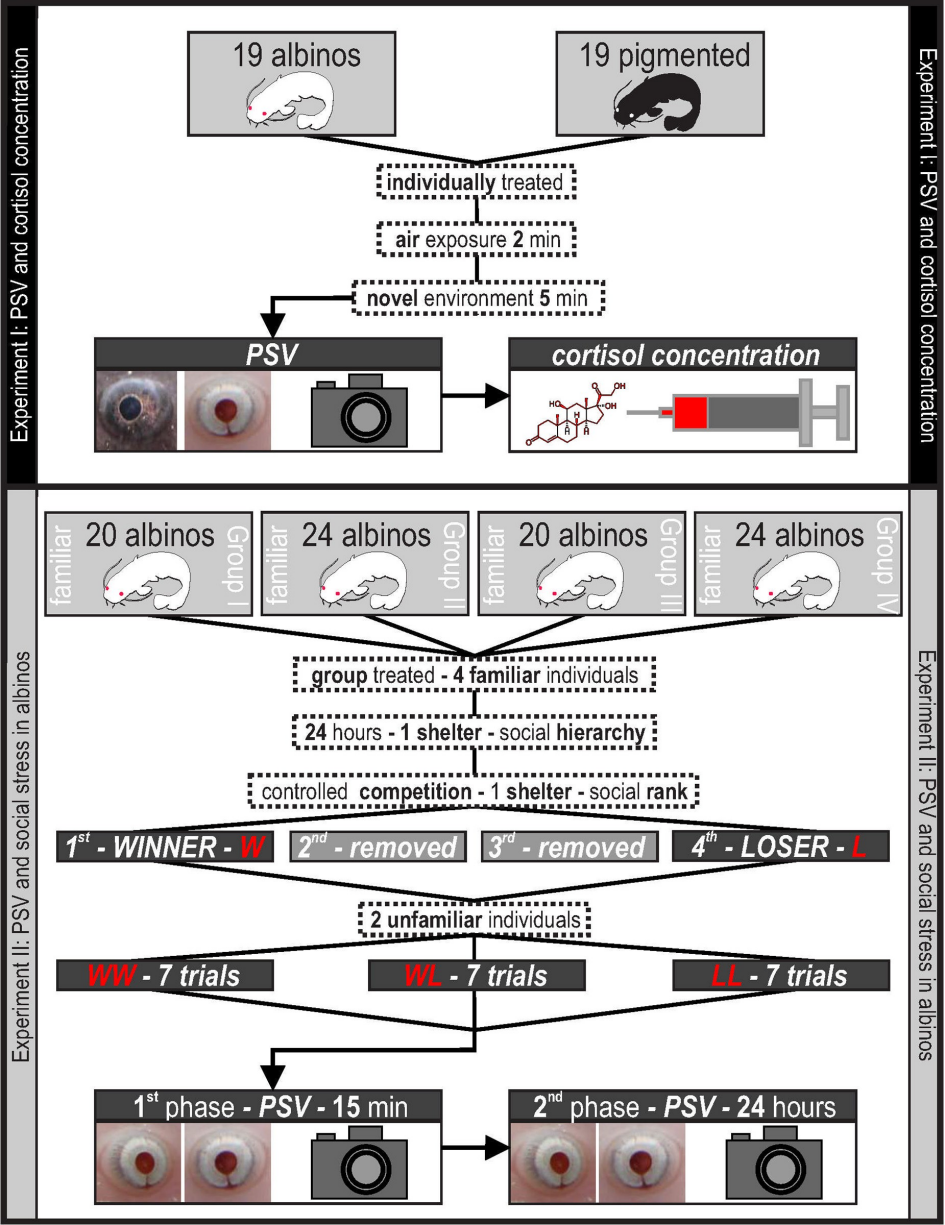
Illustrative figure of the experimental design.

The aim of our study was to validate PSV as a general stress response in fish. Therefore, we performed validation using individual pigmented and albino fish subjected to a clearly defined stressor as the first step to define the basic PSV/cortisol relationship (Experiment I). Subsequently, we aimed to test whether PSV can be used as an indicator of more complicated social stress conditions in albinos (Experiment II), for which methods based on variability in pigmentation and/or eye darkening are inapplicable [e.g. 10]. The experimental design illustrated in Fig 1.

### Experiment I: PSV and cortisol concentration

Nineteen albinos (mean weight 140 g, range 87–220 g; mean standard length 255 mm, range 208–306 mm) and nineteen pigmented (mean weight 175 g, range 93–438 g; mean standard length 270 mm, range 226–376 mm) randomly selected juvenile catfish were used to investigate the relationship between PSV and concentration of cortisol in blood plasma. To induce a stress response, we exposed fish to a combination of air exposure and novel environmental stressors. Catfish were individually netted and maintained out of water for 2 min [40] and subsequently introduced to a novel environment for 5 min (aquarium; footprint 40 x 60 cm; water level 10 cm; no shelters; based on Barreto and Volpato [41]). A set of approximately 10 photos of fish eyes was taken through the glass of the aquarium after this procedure using a Canon EOS 650D camera (18 Mpx CMOS hybrid sensor) with a macro lens (Canon Inc., Japan). The photos were taken without the flash to prevent disturbing and the camera was positioned behind the longer side glass of the aquarium. The distance of camera from the glass of the aquarium varied depending on the position of the fish; generally, the distance was in tens of centimeters. The fish showed no signs of fear or abnormal behavior such as an escape or rapid changes of movement during photography. Individuals used in the experiment were well acclimatized in the laboratory and were used to people movements behind the glass of the aquarium during daily care and feeding, so people with camera behind the glass was not that different from what they were used to on a daily basis. We obtained a blood sample from each tested individual to determine cortisol concentration immediately after taking photos. Blood was drawn from the *vena caudalis* using an 18G 1½ in syringe with heparin as anticoagulant (Heparin inj., Léčiva, Czech Republic), and samples were stabilized with 40 IU of sodium heparin per 1 ml of blood. The procedure was based on unified methods for the hematological examination of fish [57, 58]. For biochemical plasma analyses, blood was separated via centrifugation at 12,000 x g for 10 min at 4°C, and plasma samples were stored at -80°C until analysis. Cortisol concentrations were measured using a VETTEST 8008 analyzer with snap reader (IDEXX Laboratories Inc., Westbrook, ME, USA; [59, 60]) The analyzer uses dry chemical and colorimetric analysis techniques. The aquarium was thoroughly rinsed and filled with clean aged tap water before insertion of a new specimen.

### Experiment II: PSV and social stress in albinos

A total of eighty-eight albinos (mean weight, 14 g; range, 9–22 g; mean standard length 111 mm, range 93–133 mm) were used to investigate whether a penultimate experience (prior fighting/social experience according to Hsu and Wolf [46] from a contest would be reflected by a stress response during the subsequent contact. Four randomly selected familiar individuals from one storage tank were relocated into an empty storage tank of identical size. The same physical conditions, including lighting, were simulated; however, only one shelter of an identical size and shape as described for the storage tanks was included to accelerate competition among individuals. Fish were left in this tank for the following 24 hours to establish a social hierarchy. Subsequently, each group of four specimens were relocated to a novel environment (aquarium; footprint 40 x 60 cm; water level 10 cm) with only one shelter present (the same as that in the storage tank). All specimens from each group were introduced into the tank at the same time and, after the procedure described below was completed, the tank was prepared for a new group by thoroughly rinsing and filling it with clean, aged tap water. The first individual that occupied a shelter was removed from the aquarium and identified as a winner (W); the second and third occupants were removed successively, and the last, fourth individual was identified as a loser (L). Each individual was removed together with the shelter (fish was inside the shelter) using a net and shelter was then returned to the aquarium. The fish were separated individually in order to facilitate their identification and had a recovery period of 300 s before the follow-up treatment to maintain their previous winning experience [61]. Unfamiliar winners and losers from different storage tanks were subsequently placed into a set of experimental aquaria (footprint 30 x 15 cm, depth 20 cm) without shelters to form experimental pairs in the following treatment combinations: winner-winner (WW), loser-loser (LL), and winner-loser (WL). Because unfamiliar individuals were marked with different VIE colors, we were able to visually distinguish individuals in the experimental aquaria. This marking also allowed us to identify individuals in the photos of eyes taken after 15 min of occupancy in the experimental aquarium (referred to as ‘phase one’). A set of approximately 10 photos of fish eyes was taken using the same equipment used in the previous experiment. Next, the experimental pairs were left in aquaria for the following 24 h, and their eyes were photographed again (referred to as ‘phase two’). Every treatment combination (WW, LL, and WL) was repeated seven times, resulting in the use of 42 individuals for the subsequent analysis of PSV.

### Analysis of PSV

PSV was determined using digital photos that were analyzed with the help of ImageJ software [62]. The number of pixels depicting the pupil was compared to the number of pixels depicting the iris, and its ratio was used to determine the percentage of pupil dilatation. For every fish, 4 photos were analyzed from every sampling occasion. All four obtained pupil dilatation values per individual were used in further analyses to account for variability in pupil dilatation determination.

### Statistical analyses

Statistical analyses were performed using the SAS software package (SAS Institute Inc., version 9.4, www.sas.com). Mixed models with random factors were used to account for the repeated measures collected for the same experimental units (individual fish). A separate model was fitted for data from experiments I and II. PROC MIXED was used to analyze pupil dilatation in relation to cortisol blood concentration, weight, length and pigmentation (pigmented vs. albino; experiment I; model I) and in relation to the experimental phase, weight, length, previous experience and treatment category (experiment II; model II). Variables were log transformed to meet normality requirements when needed. The significance of explanatory variables was assessed using an F-test. Least-squares means (henceforth referred to as ‘adjusted means’) were subsequently computed for particular classes. The differences between classes were tested with t-tests, and a Tukey-Kramer adjustment was used for multiple comparisons. Degrees of freedom were calculated using the Kenward-Roger method [63].

## Results

### Experiment I: PSV and cortisol concentration

**Fig 2:**
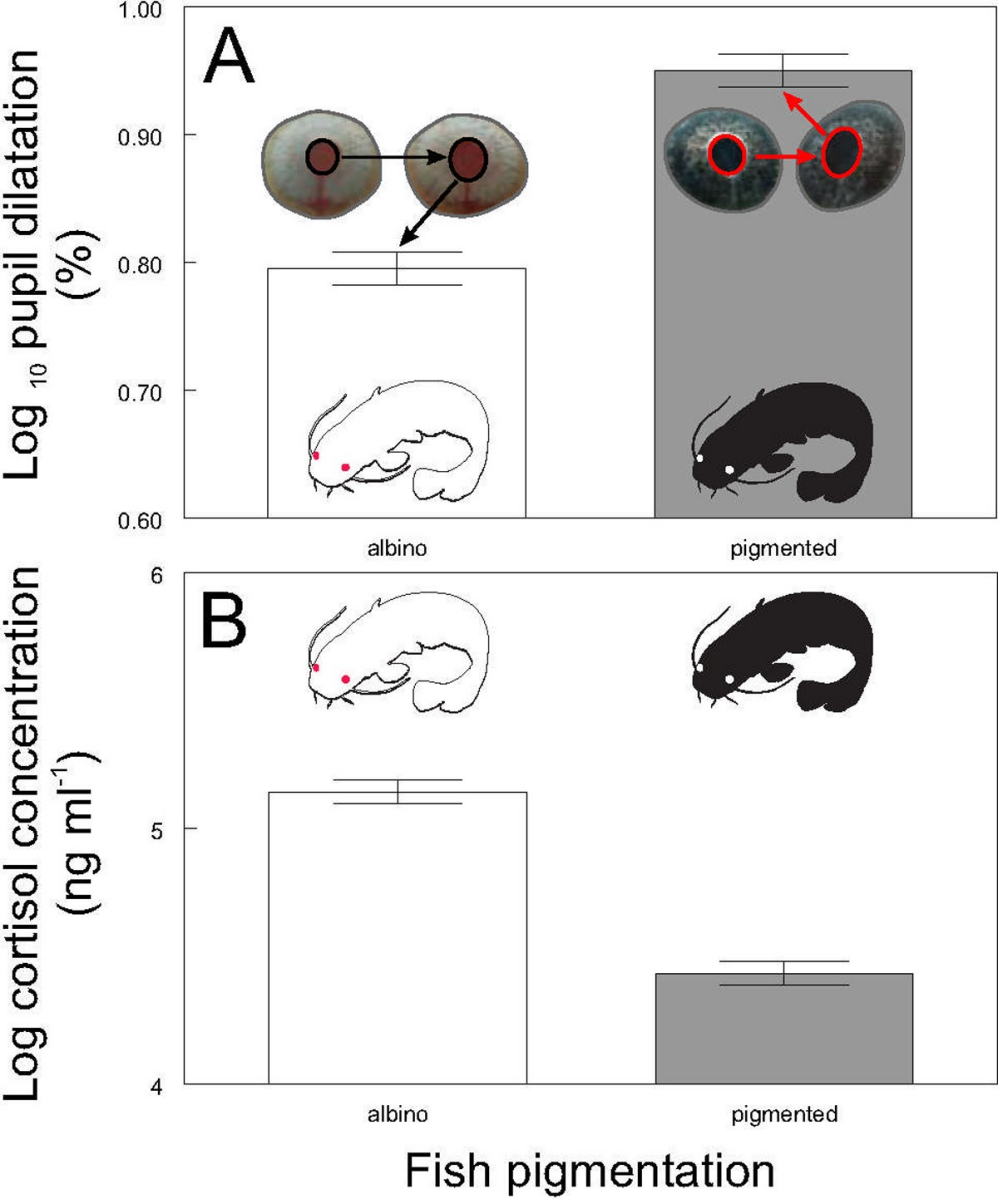
Pupil dilatation (**A**) and cortisol blood concentration (**B**) according to the fish pigmentation. Values are adjusted means +/- S.E. Different colored bars within one part of the figure indicate significant differences (Adj. P < 0.05). Part A includes illustrative picture of pupil dilatation before and after acute stress.

The mean and standard deviation of pupil dilatation of individual fish during the first experiment were 7.9% and 0.77 respectively and there was no significant relationship between pupil dilatation and body size (weight F_2, 152_ = 0.05, P ˃ 0.82; length F_2, 152_ = 0.05, P ˃ 0.83). No significant size differences were detected between the pigmented and albino catfish (weight P˃0.1, n = 38; standard length P˃0.16, n = 38). Albinos showed lower pupil dilatation (F_1, 152_ = 68.81, P < 0.0001; Fig 2A) and higher cortisol values (F_1, 38_ = 117.46, P < 0.0001; Fig 2B) than did pigmented conspecifics. Mean raw cortisol value of albino catfish was 174.91 ng / ml (range 120.36–263.97 ng / ml), while mean raw cortisol value of pigmented catfish was 85.89 ng / ml (range 50.26–120.35) ng / ml.

**Fig 3:**
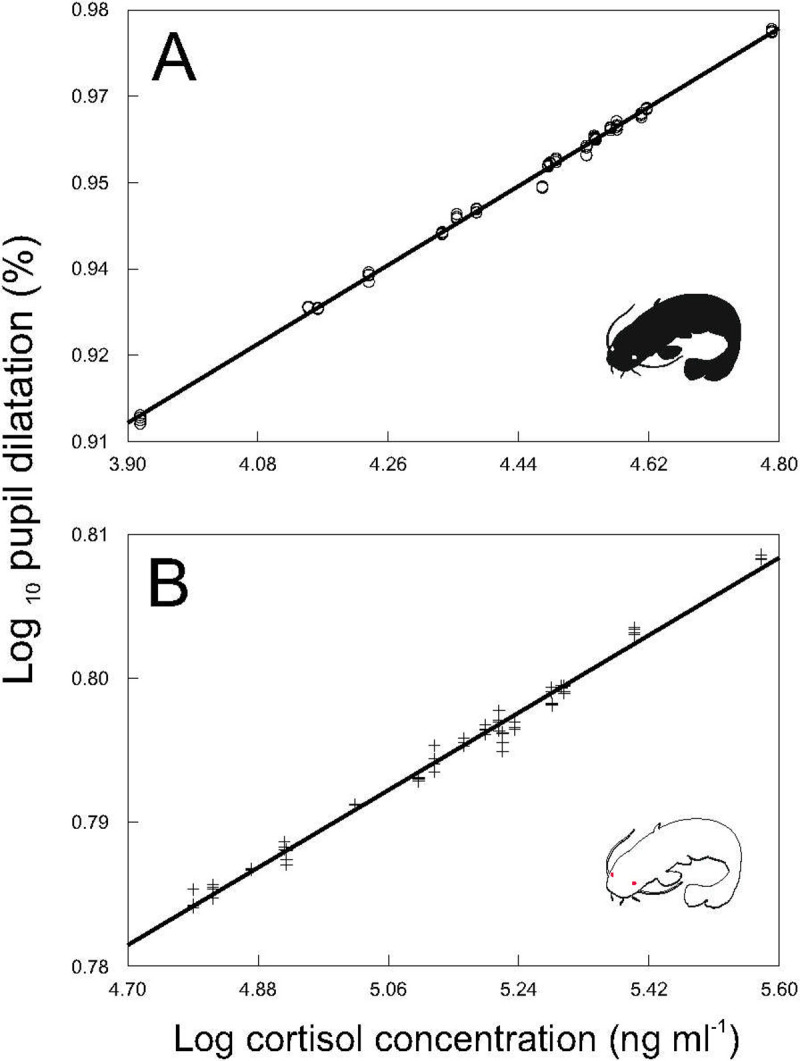
Relationship between pupil dilatation and cortisol blood concentrations. Values in pigmented (**A**; *y = 0*.*6359 + 0*.*0711x; r*^*2*^ *= 0*.*99; P < 0*.*0001*) and albino (**B**; *y = 0*.*641 + 0*.*0299x; r*^*2*^ *= 0*.*98; P < 0*.*0001*) catfish. Predicted values are from the mixed model analyses.

Our results proved that PSV could be used as a stress indicator in fish, as after exposure to a standardized stressor, catfish showed a clear positive relationship between pupil dilatation and cortisol concentrations (F_2, 152_ = 36.32, P < 0.0001). This relationship was observed for both pigmented (Fig 3A) and albino (Fig 3B) specimens, suggesting its stability irrespective of individual ability to express social communication by coloring.

### Experiment II: PSV and social stress in albinos

**Fig 4:**
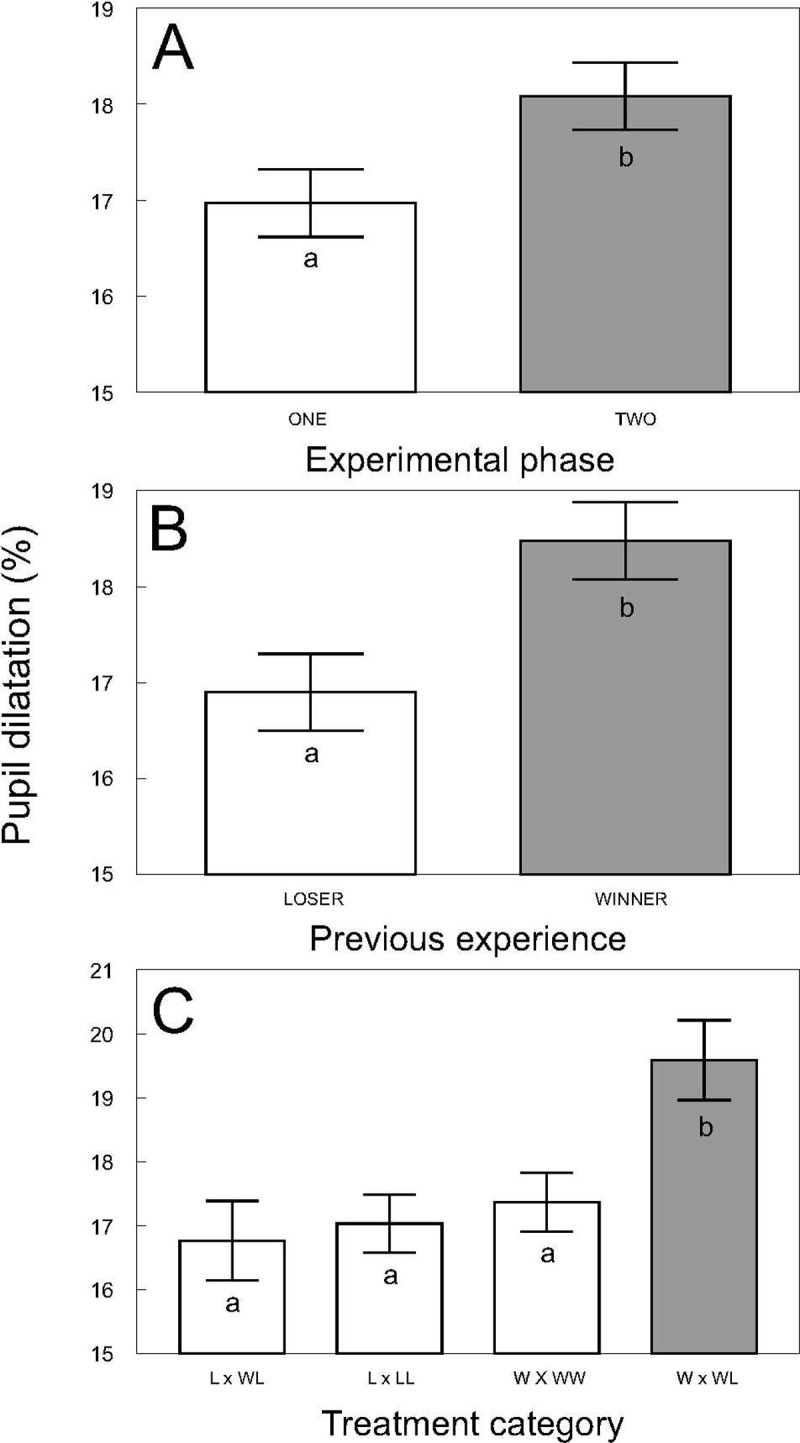
Pupil dilatation according to the experimental phase. **A**; values according to the experimental phase; ‘one’ sampled after 15 min, ‘two’ sampled after 24 h). **B**; values according to catfish previous experience (loser and winner from a penultimate contest) and **C**; values according to treatment combination (‘W x WL’ winner from the winner/loser combination, ‘W x WW’ winner from the winner/winner combination, ‘L x LL’ loser from the loser/loser combination, and ‘L x WL’ loser from the winner/loser combination). Values are adjusted means +/- S. E. Different colored and lettered bars within one part of the figure indicate mutually significant differences (Adj. P < 0.05), while differences among the same colored and lettered bars are nonsignificant (Adj. P > 0.05).

The follow-up experiment, in which the mean and standard deviation of pupil dilatation of individual fish were 16.8% and 2.76 respectively, showed that albino catfish adjusted their pupil dilatation according to the result of their previous contest as well as their subsequent contact. Pupil dilatation was generally increased after 24 h contact of two unfamiliar catfish specimens in a novel environment (F_1, 211_ = 7.15, P < 0.0081; Fig 4A), and higher pupil dilatation was observed in winners than in losers of the previous contest across both experimental phases (F_1, 275_ = 8.60, P < 0.0036; Fig 4B) with no significant relationship observed between pupil dilatation and body size (weight F_1, 99.5_ = 0.06, P ˃ 0.8; length _F1, 104_ = 2.17, P ˃ 0.14). Furthermore, the highest pupil dilatation among participants and treatment combinations was observed for the winners of WL contests, which was significantly higher than that observed in all other treatment combinations (F_2, 312_ = 4.59, P < 0.0109; Fig 4C; Adj. P < 0.05). The second highest pupil dilation was found for the winner in a WW contest, followed by the loser in an LL and the loser in a WL contest; nevertheless, differences among these treatments were nonsignificant (Adj. P > 0.05).

## Discussion

Many methodological approaches have been used to observe stress in animals, although non-invasive methods that limit the manipulation of tested individuals are becoming increasingly appreciated. The observation of PSV associated with stress and arousal in humans and mammals has been extended to stress observation in fish. The method proposed in the present study can be considered for use in laboratory experiments and/or aquaculture production (e.g., of ornamental fish) to evaluate stress level and animal welfare. The present study found that pupil dilatation and cortisol plasma concentrations were increased in fish exposed to stress conditions. These relationships were observed for both albinos and pigmented catfish. Pupil dilatation also increased following social interactions between dominant winners and submissive losers in albinos.

In our study, under stressful conditions, pigmented and albino catfish displayed pupil dilatation accompanied by an increase in cortisol levels in blood plasma. Short-term stressors such as relocation and confinement usually increase cortisol levels in blood plasma [also see 3 for a review, 64, 65]. In fish, increased cortisol concentrations are usually accompanied by other stress indicators, such as intensified gill ventilation [65] and/or subordinate behavior [2]. Both pupil dilatation and higher cortisol levels are associated with fear, stress and/or arousal, as reported in previous studies of human beings [38, 39, 66]. The present results showed a link among PSV, increased levels of cortisol and stress in fish and introduce a possible noninvasive method of stress detection in albino fish.

Our experiment showed that PSV observed during the contact of albino individuals is influenced by the results of their penultimate contest. The highest stress-induced response denoted a winner; the largest dilatation indicated a winner in the WL combination, while the lowest dilatation denoted a loser in the WL combination. The penultimate winning experience tends to modify the behavior of an individual during the next contest in which winning individuals tend to behave more aggressively towards a new contestant and their chance to win again increases (winner effect), while losers tend to behave more submissively and have a higher chance of losing again (loser effect) [67]. In addition, the results of the contest are typically reflected in the physiology of the contestants [68]. An increase in circulating androgens, e.g., testosterone hormone, predesignate winners for a victory in the next contest [69]. Hormones produced by a loser and/or the perception of winning itself can be other reasons for higher emotional arousal of an individual with penultimate winning experience [70]. Our results tended to show perceivable socially induced stress in winner and looser albinos that is reflected by PSV.

A higher stress-induced response of albino catfish with penultimate winning experience can be discussed in terms of hierarchy dominance. Higher stress among dominant individuals during contests results in a facilitated long-term approach to resources, whereas subordinate individuals are subjected to chronic stress as a result of long-term limited availability of resources. These phenomena have been reported in several vertebrates, including fish [4, 5, 71]. However, in fish, the relationship between dominancy and stress may not readily detectable. For example, no difference in acute stress between dominant and subordinate individuals occurred during cooperative breeding in cichlid *Neolamprologus pulcher* (Trewavas and Poll, 1952), while higher chronic stress was associated with dominancy, and its level in subordinate individuals depended on individual behavior, position in a social hierarchy and social stability of a group [72]. Similarly, subordinate *N*. *pulcher* females that frequently engaged in nonaggressive interactions with dominant females had lower cortisol levels, while subordinate females in groups in which the dominant breeding pair behaved antagonistically towards each had higher cortisol levels [73]. Both examples illustrate the relationship between stress stimuli and dominancy as influenced by species-specific social conditions. The relationship between penultimate winners and losers in albino catfish corresponds to the relationship between dominant and subordinate individuals wherein the higher pupil dilatation of dominant winners reflects both stress and arousal from the perception of the penultimate winning experience. Our results tended to show PSV in albino catfish as a result of socially induced stress perceptible primarily in dominant conspecifics.

Albinism can be considered a behavioral syndrome accompanied by physiological limitations, e.g., reduced adaptation to light resulting in photoreceptor degradation [74], and is often demonstrated by a reduction in vision and movement perception [75, 76], acrophobia and neophobia [77, 78]. Our results showed that albino and pigmented fish perceive stress similarly, but albinos displayed lower pupil dilatation and higher cortisol level. Lower pupil dilatation can be considered in association with degradation of the photoreceptors resulting in impair visual acuity as reported in physiological studies of rodents [79]. Higher cortisol level can be associated with the inability of albinos to inform conspecifics about their socially induced status by changes in coloring. This limitation can be reflected in behavioral performance and demonstrated by other physiological differences between pigmented and albino individuals. In salmonids, the density of melanin-based and carotenoid-based spots is reflected in behavioral performances [8, 80], e.g., individuals with more spots displayed lower endurance and oxygen consumption [81]. Accordingly, albinos displayed higher sensitivity to stress and diseases [82, 83] and/or lower aggressiveness and different behavior in a group [15]. For example, albino morph cichlid *Metriaclima zebra* (Boulenger, 1899) was more sensitive to brood parasitism of the cuckoo catfish *Synodontis multipunctatus* (Boulenger, 1898), probably due to lower aggressiveness and visual acuity [84]. In general, behavior and physiology in animals, including albinos, correspond to coloring intensity as determined by pleiotropic genetic influence [13, 85, 86]. Additionally, the results showed that socially induced stress in albinos that cannot be signaled by changes in body coloring can be detected from their pupil dilatation.

## Conclusions

Pupil dilatation and plasma cortisol increased in fish exposed to stress conditions.

The relationship was observed for both, albinos and pigmented catfish.

Noninvasive method to study stress levels in fish was introduced.

## Supporting information

S1 Table Cortisol and PSV values for the Experiment I. Fish ID, fish color (albino and pigmented fish), photo ID, cortisol concentration and PSV values.
(PDF)

S2 Table PSV for the Experiment II. Fish ID, Treatment combination, Results of the treatments, photo ID and PSV values.
(PDF)
