# Supplementary material for: Pupil size variation as a response to stress in European catfish and its application for social stress detection in albino conspecifics
Source: PLoS One. 2020 Dec 31;15(12):e0244017. doi: 10.1371/journal.pone.0244017 (PMC7775050; doi:10.1371/journal.pone.0244017)
Supplement: S1 Table — Fish ID, fish color (albino and pigmented fish), photo ID, cortisol concentration and PSV values. (PDF) [file pone.0244017.s001.pdf]

fish_ID	albino	photo_ID	cortisol	PSV
11	yes	1	4.79049	0.78404
11	yes	2	4.79049	0.78530
11	yes	3	4.79049	0.78423
11	yes	4	4.79049	0.78417
12	yes	1	5.21298	0.79775
12	yes	2	5.21298	0.79696
12	yes	3	5.21298	0.79633
12	yes	4	5.21298	0.79704
13	yes	1	5.29957	0.79949
13	yes	2	5.29957	0.79930
13	yes	3	5.29957	0.79933
13	yes	4	5.29957	0.79931
14	yes	1	5.28614	0.79809
14	yes	2	5.28614	0.79815
14	yes	3	5.28614	0.79813
14	yes	4	5.28614	0.79825
15	yes	1	5.57584	0.80858
15	yes	2	5.57584	0.80824
15	yes	3	5.57584	0.80832
15	yes	4	5.57584	0.80825
16	yes	1	4.87091	0.78676
16	yes	2	4.87091	0.78673
16	yes	3	4.87091	0.78664
16	yes	4	4.87091	0.78675
17	yes	1	5.01383	0.79115
17	yes	2	5.01383	0.79120
17	yes	3	5.01383	0.79117
17	yes	4	5.01383	0.79119
18	yes	1	5.10182	0.79300
18	yes	2	5.10182	0.79282
18	yes	3	5.10182	0.79295
18	yes	4	5.10182	0.79308
19	yes	1	4.81745	0.78563
19	yes	2	4.81745	0.78531
19	yes	3	4.81745	0.78549
19	yes	4	4.81745	0.78469
20	yes	1	5.28564	0.79907
20	yes	2	5.28564	0.79936
20	yes	3	5.28564	0.79902
20	yes	4	5.28564	0.79903
21	yes	1	5.40060	0.80349
21	yes	2	5.40060	0.80339
21	yes	3	5.40060	0.80301
21	yes	4	5.40060	0.80316
22	yes	1	4.91954	0.78737
22	yes	2	4.91954	0.78809
22	yes	3	4.91954	0.78799

22	yes	4	4.91954	0.78700
33	yes	1	5.16450	0.79581
33	yes	2	5.16450	0.79527
33	yes	3	5.16450	0.79550
33	yes	4	5.16450	0.79528
34	yes	1	5.21792	0.79489
34	yes	2	5.21792	0.79621
34	yes	3	5.21792	0.79552
34	yes	4	5.21792	0.79616
36	yes	1	4.91618	0.78827
36	yes	2	4.91618	0.78861
36	yes	3	4.91618	0.78808
36	yes	4	4.91618	0.78809
37	yes	1	5.12384	0.79404
37	yes	2	5.12384	0.79345
37	yes	3	5.12384	0.79531
37	yes	4	5.12384	0.79440
38	yes	1	5.19418	0.79608
38	yes	2	5.19418	0.79641
38	yes	3	5.19418	0.79645
38	yes	4	5.19418	0.79675
39	yes	1	5.30321	0.79948
39	yes	2	5.30321	0.79940
39	yes	3	5.30321	0.79894
39	yes	4	5.30321	0.79902
40	yes	1	5.23453	0.79643
40	yes	2	5.23453	0.79694
40	yes	3	5.23453	0.79657
40	yes	4	5.23453	0.79654
1	no	1	3.91721	0.91390
1	no	2	3.91721	0.91293
1	no	3	3.91721	0.91427
1	no	4	3.91721	0.91345
2	no	1	4.14962	0.93171
2	no	2	4.14962	0.93187
2	no	3	4.14962	0.93171
2	no	4	4.14962	0.93169
3	no	1	4.35478	0.94617
3	no	2	4.35478	0.94690
3	no	3	4.35478	0.94645
3	no	4	4.35478	0.94645
4	no	1	4.23324	0.93694
4	no	2	4.23324	0.93743
4	no	3	4.23324	0.93709
4	no	4	4.23324	0.93594
5	no	1	4.79040	0.97691
5	no	2	4.79040	0.97656
5	no	3	4.79040	0.97638

5	no	4	4.79040	0.97660
6	no	1	4.38215	0.94767
6	no	2	4.38215	0.94722
6	no	3	4.38215	0.94715
6	no	4	4.38215	0.94780
7	no	1	4.49223	0.95533
7	no	2	4.49223	0.95566
7	no	3	4.49223	0.95594
7	no	4	4.49223	0.95591
8	no	1	4.61026	0.96262
8	no	2	4.61026	0.96295
8	no	3	4.61026	0.96300
8	no	4	4.61026	0.96325
9	no	1	4.16278	0.93150
9	no	2	4.16278	0.93168
9	no	3	4.16278	0.93163
9	no	4	4.16278	0.93156
10	no	1	4.33480	0.94398
10	no	2	4.33480	0.94379
10	no	3	4.33480	0.94371
10	no	4	4.33480	0.94365
23	no	1	4.48357	0.95516
23	no	2	4.48357	0.95526
23	no	3	4.48357	0.95510
23	no	4	4.48357	0.95511
24	no	1	4.54701	0.95893
24	no	2	4.54701	0.95933
24	no	3	4.54701	0.95904
24	no	4	4.54701	0.95917
25	no	1	4.61730	0.96390
25	no	2	4.61730	0.96393
25	no	3	4.61730	0.96415
25	no	4	4.61730	0.96404
26	no	1	4.56768	0.96100
26	no	2	4.56768	0.96054
26	no	3	4.56768	0.96084
26	no	4	4.56768	0.96086
27	no	1	4.47301	0.95131
27	no	2	4.47301	0.95119
27	no	3	4.47301	0.95126
27	no	4	4.47301	0.95137
28	no	1	4.54489	0.95931
28	no	2	4.54489	0.95960
28	no	3	4.54489	0.95924
28	no	4	4.54489	0.95934
29	no	1	4.48119	0.95482
29	no	2	4.48119	0.95490
29	no	3	4.48119	0.95462

29	no	4	4.48119	0.95483
30	no	1	4.53421	0.95650
30	no	2	4.53421	0.95770
30	no	3	4.53421	0.95644
30	no	4	4.53421	0.95798
31	no	1	.	.
31	no	2	.	.
31	no	3	.	.
32	no	1	4.57626	0.96107
32	no	2	4.57626	0.96131
32	no	3	4.57626	0.96060
32	no	4	4.57626	0.96195
