# Supplementary material for: Pupil size variation as a response to stress in European catfish and its application for social stress detection in albino conspecifics
Source: PLoS One. 2020 Dec 31;15(12):e0244017. doi: 10.1371/journal.pone.0244017 (PMC7775050; doi:10.1371/journal.pone.0244017)
Supplement: S2 Table — Fish ID, Treatment combination, Results of the treatments, photo ID and PSV values. (PDF) [file pone.0244017.s002.pdf]

fish_ID	treatment	win_los	photo_ID	phase
1	WL	W	1.	1
1	WL	W	2.	1
1	WL	W	3.	1
1	WL	W	4.	1
1	WL	W	1.	2
1	WL	W	2.	2
1	WL	W	3.	2
1	WL	W	4.	2
1	WL	L	1.	1
1	WL	L	2.	1
1	WL	L	3.	1
1	WL	L	4.	1
1	WL	L	1.	2
1	WL	L	2.	2
1	WL	L	3.	2
1	WL	L	4.	2
3	WL	W	1.	1
3	WL	W	2.	1
3	WL	W	3.	1
3	WL	W	4.	1
3	WL	W	1.	2
3	WL	W	2.	2
3	WL	W	3.	2
3	WL	W	4.	2
3	WL	L	1.	1
3	WL	L	2.	1
3	WL	L	3.	1
3	WL	L	4.	1
3	WL	L	1.	2
3	WL	L	2.	2
3	WL	L	3.	2
3	WL	L	4.	2
4	WW	W	1.	1
4	WW	W	2.	1
4	WW	W	3.	1
4	WW	W	4.	1
4	WW	W	1.	2
4	WW	W	2.	2
4	WW	W	3.	2
4	WW	W	4.	2
4	WW	W	1.	1
4	WW	W	2.	1
4	WW	W	3.	1
4	WW	W	4.	1
4	WW	W	1.	2
4	WW	W	2.	2
4	WW	W	3.	2

4	WW	W	4.	2
5	WL	W	1.	1
5	WL	W	2.	1
5	WL	W	3.	1
5	WL	W	4.	1
5	WL	W	1.	2
5	WL	W	2.	2
5	WL	W	3.	2
5	WL	W	4.	2
5	WL	L	1.	1
5	WL	L	2.	1
5	WL	L	3.	1
5	WL	L	4.	1
5	WL	L	1.	2
5	WL	L	2.	2
5	WL	L	3.	2
5	WL	L	4.	2
6	WL	W	1.	1
6	WL	W	2.	1
6	WL	W	3.	1
6	WL	W	4.	1
6	WL	W	1.	2
6	WL	W	2.	2
6	WL	W	3.	2
6	WL	W	4.	2
6	WL	L	1.	1
6	WL	L	2.	1
6	WL	L	3.	1
6	WL	L	4.	1
6	WL	L	1.	2
6	WL	L	2.	2
6	WL	L	3.	2
6	WL	L	4.	2
7	WL	W	1.	1
7	WL	W	2.	1
7	WL	W	3.	1
7	WL	W	4.	1
7	WL	W	1.	2
7	WL	W	2.	2
7	WL	W	3.	2
7	WL	W	4.	2
7	WL	L	1.	1
7	WL	L	2.	1
7	WL	L	3.	1
7	WL	L	4.	1
7	WL	L	1.	2
7	WL	L	2.	2
7	WL	L	3.	2

7	WL	L	4.	2
23	LL	L	1.	1
23	LL	L	2.	1
23	LL	L	3.	1
23	LL	L	4.	1
23	LL	L	1.	2
23	LL	L	2.	2
23	LL	L	3.	2
23	LL	L	4.	2
23	LL	L	1.	1
23	LL	L	2.	1
23	LL	L	3.	1
23	LL	L	4.	1
23	LL	L	1.	2
23	LL	L	2.	2
23	LL	L	3.	2
23	LL	L	4.	2
8	WW	W	1.	1
8	WW	W	2.	1
8	WW	W	3.	1
8	WW	W	4.	1
8	WW	W	1.	2
8	WW	W	2.	2
8	WW	W	3.	2
8	WW	W	4.	2
8	WW	W	1.	1
8	WW	W	2.	1
8	WW	W	3.	1
8	WW	W	4.	1
8	WW	W	1.	2
8	WW	W	2.	2
8	WW	W	3.	2
8	WW	W	4.	2
9	WW	W	1.	1
9	WW	W	2.	1
9	WW	W	3.	1
9	WW	W	4.	1
9	WW	W	1.	2
9	WW	W	2.	2
9	WW	W	3.	2
9	WW	W	4.	2
9	WW	W	1.	1
9	WW	W	2.	1
9	WW	W	3.	1
9	WW	W	4.	1
9	WW	W	1.	2
9	WW	W	2.	2
10	WW	W	1.	1

10	WW	W	2.	1
10	WW	W	3.	1
10	WW	W	4.	1
10	WW	W	1.	2
10	WW	W	2.	2
10	WW	W	3.	2
10	WW	W	4.	2
10	WW	W	1.	1
10	WW	W	2.	1
10	WW	W	3.	1
10	WW	W	4.	1
10	WW	W	1.	2
10	WW	W	2.	2
10	WW	W	3.	2
10	WW	W	4.	2
11	WW	W	1.	1
11	WW	W	2.	1
11	WW	W	3.	1
11	WW	W	4.	1
11	WW	W	1.	2
11	WW	W	2.	2
11	WW	W	3.	2
11	WW	W	4.	2
11	WW	W	1.	1
11	WW	W	2.	1
11	WW	W	3.	1
11	WW	W	4.	1
11	WW	W	1.	2
11	WW	W	2.	2
11	WW	W	3.	2
11	WW	W	4.	2
12	LL	L	1.	1
12	LL	L	2.	1
12	LL	L	3.	1
12	LL	L	4.	1
12	LL	L	1.	2
12	LL	L	2.	2
12	LL	L	3.	2
12	LL	L	4.	2
12	LL	L	1.	1
12	LL	L	2.	1
12	LL	L	3.	1
12	LL	L	4.	1
12	LL	L	1.	2
12	LL	L	2.	2
12	LL	L	3.	2
12	LL	L	4.	2
13	LL	L	1.	1

13	LL	L	2.	1
13	LL	L	3.	1
13	LL	L	4.	1
13	LL	L	1.	2
13	LL	L	2.	2
13	LL	L	3.	2
13	LL	L	4.	2
13	LL	L	1.	1
13	LL	L	2.	1
13	LL	L	3.	1
13	LL	L	4.	1
13	LL	L	1.	2
13	LL	L	2.	2
13	LL	L	3.	2
13	LL	L	4.	2
14	LL	L	1.	1
14	LL	L	2.	1
14	LL	L	3.	1
14	LL	L	4.	1
14	LL	L	1.	2
14	LL	L	2.	2
14	LL	L	3.	2
14	LL	L	4.	2
14	LL	L	1.	1
14	LL	L	2.	1
14	LL	L	3.	1
14	LL	L	4.	1
14	LL	L	1.	2
14	LL	L	2.	2
14	LL	L	3.	2
14	LL	L	4.	2
15	WL	W	1.	1
15	WL	W	2.	1
15	WL	W	3.	1
15	WL	W	4.	1
15	WL	W	1.	2
15	WL	W	2.	2
15	WL	W	3.	2
15	WL	W	4.	2
15	WL	L	1.	1
15	WL	L	2.	1
15	WL	L	3.	1
15	WL	L	4.	1
15	WL	L	1.	2
15	WL	L	2.	2
15	WL	L	3.	2
15	WL	L	4.	2
16	WL	W	1.	1

16	WL	W	2.	1
16	WL	W	3.	1
16	WL	W	4.	1
16	WL	W	1.	2
16	WL	W	2.	2
16	WL	W	3.	2
16	WL	W	4.	2
16	WL	L	1.	1
16	WL	L	2.	1
16	WL	L	3.	1
16	WL	L	4.	1
16	WL	L	1.	2
16	WL	L	2.	2
16	WL	L	3.	2
16	WL	L	4.	2
17	WW	W	1.	1
17	WW	W	2.	1
17	WW	W	3.	1
17	WW	W	4.	1
17	WW	W	1.	2
17	WW	W	2.	2
17	WW	W	3.	2
17	WW	W	4.	2
17	WW	W	1.	1
17	WW	W	2.	1
17	WW	W	3.	1
17	WW	W	4.	1
17	WW	W	1.	2
17	WW	W	2.	2
17	WW	W	3.	2
17	WW	W	4.	2
18	LL	L	1.	1
18	LL	L	2.	1
18	LL	L	3.	1
18	LL	L	4.	1
18	LL	L	1.	2
18	LL	L	2.	2
18	LL	L	3.	2
18	LL	L	4.	2
18	LL	L	1.	1
18	LL	L	2.	1
18	LL	L	3.	1
18	LL	L	4.	1
18	LL	L	1.	2
18	LL	L	2.	2
18	LL	L	3.	2
18	LL	L	4.	2
19	WW	W	1.	1

19	WW	W	2.	1
19	WW	W	3.	1
19	WW	W	4.	1
19	WW	W	1.	2
19	WW	W	2.	2
19	WW	W	3.	2
19	WW	W	4.	2
19	WW	W	1.	1
19	WW	W	2.	1
19	WW	W	3.	1
19	WW	W	4.	1
19	WW	W	1.	2
19	WW	W	2.	2
19	WW	W	3.	2
19	WW	W	4.	2
20	LL	L	1.	1
20	LL	L	2.	1
20	LL	L	3.	1
20	LL	L	4.	1
20	LL	L	1.	2
20	LL	L	2.	2
20	LL	L	3.	2
20	LL	L	4.	2
20	LL	L	1.	1
20	LL	L	2.	1
20	LL	L	3.	1
20	LL	L	4.	1
20	LL	L	1.	2
20	LL	L	2.	2
20	LL	L	3.	2
20	LL	L	4.	2
22	LL	L	1.	1
22	LL	L	2.	1
22	LL	L	3.	1
22	LL	L	4.	1
22	LL	L	1.	2
22	LL	L	2.	2
22	LL	L	3.	2
22	LL	L	4.	2
22	LL	L	1.	1
22	LL	L	2.	1
22	LL	L	3.	1
22	LL	L	4.	1
22	LL	L	1.	2
22	LL	L	2.	2
22	LL	L	3.	2
22	LL	L	4.	2
9	WW	W	3.	2

9	WW	W	4.	2

PSV
19.7059
21.9832
23.5762
20.3648
20.8178
23.0951
24.6880
21.4767
17.0211
17.9476
15.1340
15.5296
18.1330
19.0595
16.2459
16.6415
18.7123
18.9142
20.2137
19.6071
19.8242
20.0261
21.3256
20.7190
17.4945
16.6119
16.9914
17.0110
18.6064
17.7237
18.1033
18.1229
18.7165
16.7392
17.7033
17.8824
19.8283
17.8510
18.8151
18.9943
19.0262
19.4965
18.6793
17.6088
20.1381
20.6084
19.7912

18.7207
20.3848
20.2459
18.8824
18.0006
21.4966
21.3577
19.9943
19.1125
17.8866
19.3261
15.4801
14.6063
18.9984
20.4380
16.5920
15.7182
21.6386
20.6059
20.1616
18.2365
22.7505
21.7178
21.2735
19.3484
17.0996
18.4013
17.6034
17.6737
18.2115
19.5131
18.7153
18.7856
21.8708
20.2732
19.8433
19.7869
22.9827
21.3851
20.9552
20.8988
15.5582
16.1035
14.6396
15.0340
16.6700
17.2154
15.7515

16.1458
16.2468
16.6976
16.6314
17.2654
17.3587
17.8095
17.7433
18.3772
17.5064
15.4628
15.7437
14.9204
18.6183
16.5747
16.8556
16.0323
13.5256
13.7397
14.5467
14.6227
14.6374
14.8516
15.6586
15.7346
16.0936
16.0475
16.0657
16.3964
17.2054
17.1593
17.1776
17.5083
16.5387
16.1592
16.5727
17.9913
17.6506
17.2711
17.6846
19.1031
17.5603
17.4777
16.4396
16.3817
18.6722
18.5896
16.4099

15.3324
16.1914
16.0826
17.5217
16.4443
17.3033
17.1945
16.4926
16.6945
17.9940
17.3874
17.6045
17.8064
19.1059
18.4993
16.0591
15.2244
16.2845
15.9403
17.1710
16.3363
17.3964
17.0522
15.4760
16.6973
15.9319
16.0802
16.5879
17.8092
17.0438
17.1921
17.7610
16.8784
17.2579
17.2775
18.8729
17.9902
18.3698
18.3894
14.1463
13.5758
13.7114
13.8085
15.2581
14.6876
14.8233
14.9204
19.3159

17.7183
17.2884
17.2320
20.4277
18.8301
18.4003
18.3439
16.2049
16.7413
15.5779
15.1496
17.3168
17.8532
16.6898
16.2615
15.9736
16.2102
16.0661
17.3503
17.0855
17.3221
17.1780
18.4622
16.9034
17.4789
17.6668
16.4665
18.0153
18.5908
18.7787
17.5784
17.1084
16.5834
16.3977
16.9995
18.2202
17.6953
17.5096
18.1113
13.8797
13.3093
13.4449
13.5420
14.9916
14.4211
14.5568
14.6539
18.3796

18.9250
17.4610
17.8554
19.4915
20.0368
18.5729
18.9673
15.8081
14.7306
15.5897
15.4808
16.9200
15.8425
16.7015
16.5927
19.4189
18.3862
17.9419
16.0168
20.5308
19.4981
19.0538
17.1287
16.0936
16.0475
16.0657
16.3964
17.2054
17.1593
17.1776
17.5083
17.6439
17.2428
17.0305
17.0362
18.7558
18.3547
18.1424
18.1481
15.7897
15.3026
16.1187
16.0369
16.9016
16.4145
17.2306
17.1488
16.5967

16.8683
17.0608
17.1030
17.7086
17.9802
18.1727
18.2149
17.2386
17.8141
18.0020
16.8017
18.3505
18.9260
19.1139
17.9136
13.1903
13.4045
14.2114
14.2875
14.3022
14.5164
15.3233
15.3993
15.8777
16.2963
17.1647
16.4289
16.9896
17.4082
18.2766
17.5408
15.9736
16.2102
16.0661
17.3503
17.0855
17.3221
17.1780
18.4622
16.4703
17.1968
16.0741
16.7224
17.5822
18.3087
17.1859
17.8343
17.5515

17.4935
